# Novel CAF-identifiers via transcriptomic and protein level analysis in HNSC patients

**DOI:** 10.1038/s41598-023-40908-w

**Published:** 2023-08-25

**Authors:** Nehanjali Dwivedi, Nidhi Shukla, K. M. Prathima, Manjula Das, Sujan K. Dhar

**Affiliations:** 1 Molecular Immunology, Mazumdar Shaw Medical Foundation, Narayana Health City, Bommasandra, Bangalore, Karnataka 560099 India; 2 MAHE, Manipal, 576104 India; 3 Manipal Hospital, Miller’s Road, Bangalore, Karnataka 560052 India; 4 Computational Biology, Mazumdar Shaw Medical Foundation, Narayana Health City, Bommasandra, Bangalore, Karnataka 560099 India

**Keywords:** Cancer, Computational biology and bioinformatics

## Abstract

Cancer-associated fibroblasts (CAFs), a prominent component of the tumor microenvironment, play an important role in tumor development, invasion, and drug resistance. The expression of distinct “CAF-markers” which separates CAFs from normal fibroblasts and epithelial cells, have traditionally been used to identify them. These commonly used CAF-markers have been reported to differ greatly across different CAF subpopulations, even within a cancer type. Using an unbiased -omic approach from public data and in-house RNAseq data from patient derived novel CAF cells, TIMP-1, SPARC, COL1A2, COL3A1 and COL1A1 were identified as potential CAF-markers by differential gene expression analysis using publicly available single cell sequencing data and in-house RNAseq data to distinguish CAF populations from tumor epithelia and normal oral fibroblasts. Experimental validation using qPCR and immunofluorescence revealed CAF-specific higher expression of TIMP-1 and COL1A2 as compared to other markers in 5 novel CAF cells, derived from patients of diverse gender, habits and different locations of head and neck squamous cell carcinoma (HNSC). Upon immunohistochemical (IHC) analysis of FFPE blocks however, COL1A2 showed better differential staining between tumor epithelia and tumor stroma. Similar data science driven approach utilizing single cell sequencing and RNAseq data from stabilized CAFs can be employed to identify CAF-markers in various cancers.

## Introduction

Tumor microenvironment (TME) consists of dynamic cell populations such as fibroblasts, immune cells, inflammatory cells, adipocytes, endothelial cells, mesenchymal cells, and extracellular matrix (ECM)^1–3^. Cross-talks between CAF and TME signal the tumor to survive and advance^4,5^. During cancer development, there is a prominent expansion of quiescent fibroblasts residing in the host tissue in response to the developing neoplasm^6^. CAFs can be assertively defined as cells with an elongated morphology and negative for epithelial (EpCAM), endothelial (CD31) and leukocyte (CD45) markers^7,8^.

Multiple markers have been described to define CAFs for years. For example, Fibroblast Activation Protein (FAP) and Smooth Muscle Actin (alpha SMA/ACTA2) have been employed as a marker of activated CAFs in colorectal and breast cancer respectively^8–10^, because of their high expression in the tumor stroma. However, a number of investigations have revealed that epithelial cells undergoing epithelial-mesenchymal transition (EMT) also exhibit higher levels of FAP^11^, whereas, alpha SMA displays fluctuating expression amongst various CAF subtypes^12,13^. Although not significantly upregulated in CAF populations, due to a more stable expression, not sensitive to environmental variables like hypoxia, PDGFRα and PDGFRβ have been considered as CAF-markers in breast cancer^14^. Expression of FSP1^15^, Transgelin (TAGLN) and Periostin (POSTN)^16^ in CAFs vary among CAF-subtypes as reported in colorectal cancer^13^. Podoplanin (PDPN), a membrane-bound marker that has been utilised to identify pro-tumorigenic fibroblast subpopulations lacks specificity since it is also expressed in epithelial tumor cells and inflammatory macrophages^17,18^. Integrin 11 (ITGA11) has been identified to be upregulated in CAFs from non-small cell lung cancer^19,20^, though demonstrated to be present in a variety of tumor cell lines and sensitive to hypoxia^21^ and TGF- signalling^22^, playing a dual role of an inducer and antagonist of specific CAF subtypes^23^. Though described a few years back, Microfibril Associated Protein 5 (MFAP5) and Collagen Type XI Alpha I Chain (COL11A1) have not yet found popularity among the users. Further research is therefore necessary to characterise these markers' activity and expression in various CAF subtypes^24,25^.

Not only in identifying them, CAF-markers can be utilized to classify CAFs into various subtypes in different cancers^26,27^, giving insight about their functional diversity^28–30^. For example, Interleukin-6 (IL6) has been used in pancreatic ductal adenocarcinoma (PDAC) to describe inflammatory CAFs (iCAFs). Similarly, in breast cancer, podoplanin and S100A^31^, in colorectal cancer (CRC), ACTA2-TAGLN-PDGFA, and DCN-MMP2-COL1A2^13^ have been used.

In HNSC specifically, Vimentin and ACTA2 are the most frequently used CAF-markers. Vimentin's ubiquitous expression in the whole fibroblast population as well as multiple other cell types, including macrophages, adipocytes, and the cells undergoing EMT severely limits its utility as a CAF-specific marker^32–34^. Similarly, ACTA2 has inconsistent expression across different subtypes of CAFs^12,13^. Fibronectin 1 and THY1 have been known to recognize myofibroblasts and fibroblasts of the reticular lineage respectively^35^. Two other markers—SHH and GLI1 have also been reported, however, without any exclusivity to CAFs. Many authors used highly sensitive single cell sequencing approach to classify CAFs based on differential expression of several markers in HNSC (supplementary table I). Puram et.al, identified immediate early response genes (e.g. JUN, FOS), mesenchymal markers (e.g. VIM, THY1), ligands and receptors (e.g. FGF7, TGFBR2/3), and ECM proteins (e.g. MMP11, CAV1)^36^ to classify CAFs into CAF1 and CAF2 subtypes. Wang et.al reported FTH1, TM4SF1, SLC16A3 and IER3 as markers for prognosis-related subtype of CAFs^37^. However, their functional utility and confirmation at the protein expression level have not been explored yet^36,37^.

Since many positive CAF-markers utilised lack of specificity, negative selection is commonly used to filter out the cell types that are frequently present in tumor microenvironment. In order to distinguish between epithelial and smooth muscle cells, markers like epithelium cell adhesion molecule (EpCAM) and Smoothelin (SMTN) are frequently utilised^33,38,39^. Leukocytes and endothelial cells are typically excluded using CD45, CD34, and CD11b^40^.

Though not directly for the clinic, CAF-markers are very important for research, to differentiate them from the tumor cells to study the mechanism of action of the microenvironment in cancer progression. Multiple methods, as in Supplementary Table II, have been employed to arrive at a set of CAF-markers to distinguish them from normal fibroblast and epithelial cells. Since the development of sequencing technologies, availability of multi-omics data has transformed medicine and biology by allowing integrated system-level techniques to be developed^41^. Researchers may compare, compute, and analyse the expression patterns of multiple genes across samples and cell types using gene expression analyses methods such as microarray, mRNA sequencing, and single cell sequencing that are more robust as compared to the gold standard immunohistochemistry (IHC) methodology. However, experimental validation of the identified markers on pure primary cultures becomes indispensable to support the omics data in localization studies. Thus, it is important to consider all these aspects to analyse the markers before reporting.

The aim of the study was therefore, to explore reliable CAF-marker(s) via multi-omics analysis followed by experimental validation in HNSC, the sixth most prevalent cancer in the world, affecting the mucosal epithelium of the larynx, oral cavity, and pharynx^42^.

The present study employed an integrative analysis of publicly available single-cell, bulk gene expression data and in-house RNAseq data to identify the CAF-markers, which were then validated on patient derived cells by qPCR, immunofluorescence and on HNSC-FFPE blocks by IHC.

## Results

### Identification of differentially expressed genes between CAF and epithelial cells

**Figure 1:**
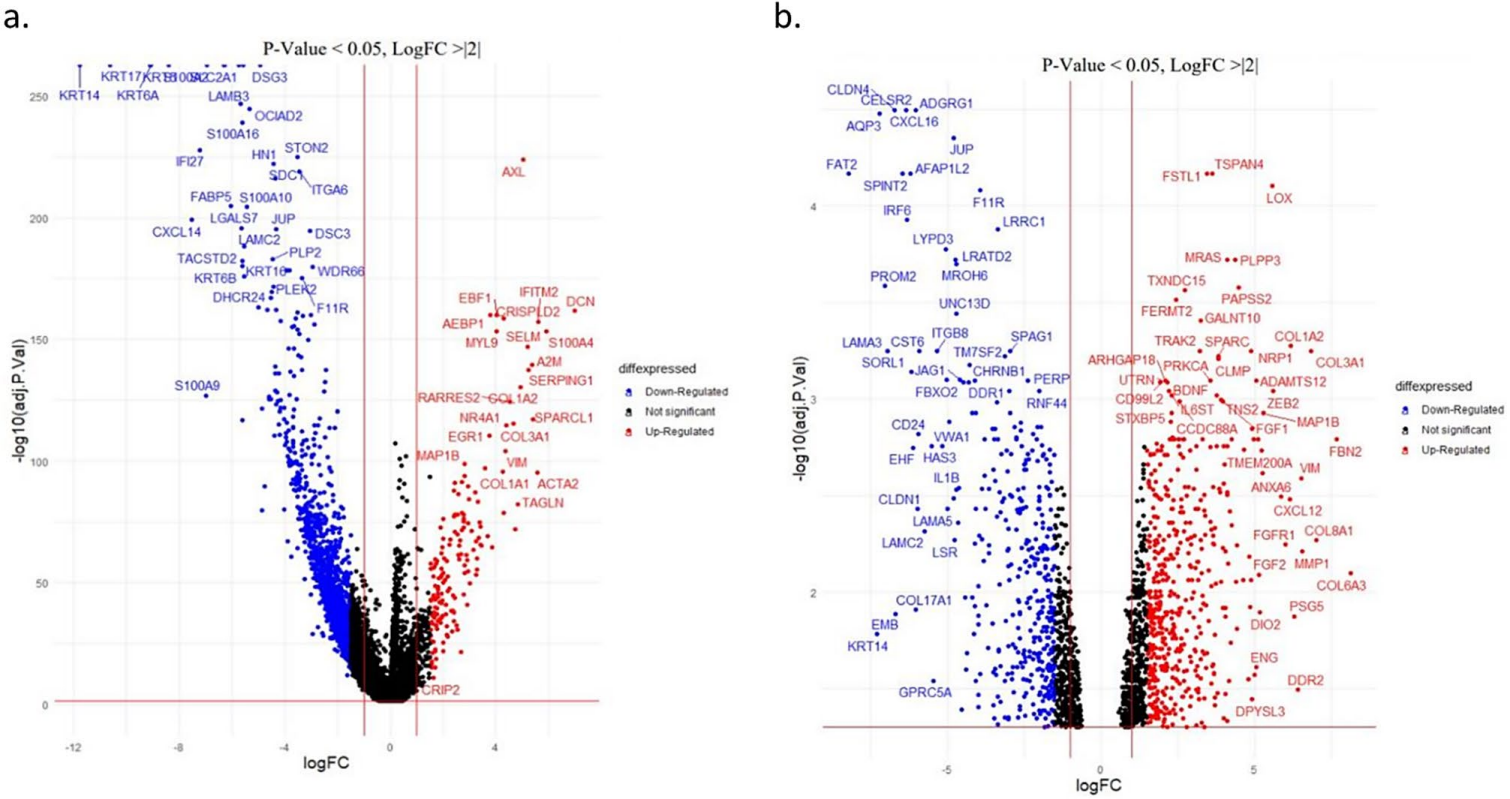
Differentially expressed genes between CAF and epithelial cells﻿. (**a**) from in-house RNAseq data from patient derived CAF (MhCT08-F and MhCT12-F) and epithelial cell lines (MhCT08-E and MhCT12-E) (**b**) Single cell sequencing data from GSE10332237^36^. Overexpressed genes (logFoldChange >|2| and adjusted *p*-value < 0.05) in fibroblasts and epithelial cells are shown as red and blue dots respectively.

Differential gene expression analysis between theestablished CAFs MhCT012-F or MhCT08-F and epithelials MhCT08-E and MhCT12-E revealed 313 significantly upregulated genes (Fig. 1a), whereas single-cell sequencing data from non-lymphatic fibroblast and epithelial cells obtained from GSE103322 [21] revealed 97 genes that were up-regulated in CAFs as compared to epithelial cells (Fig. 1b). Twenty two genes were concordant between the two differential expression subsets (Supplementary Table III) that shows eight genes having significant p-values between CAFs vs normal fibroblasts.

### Selection of putative CAF-markers

**Figure 2:**
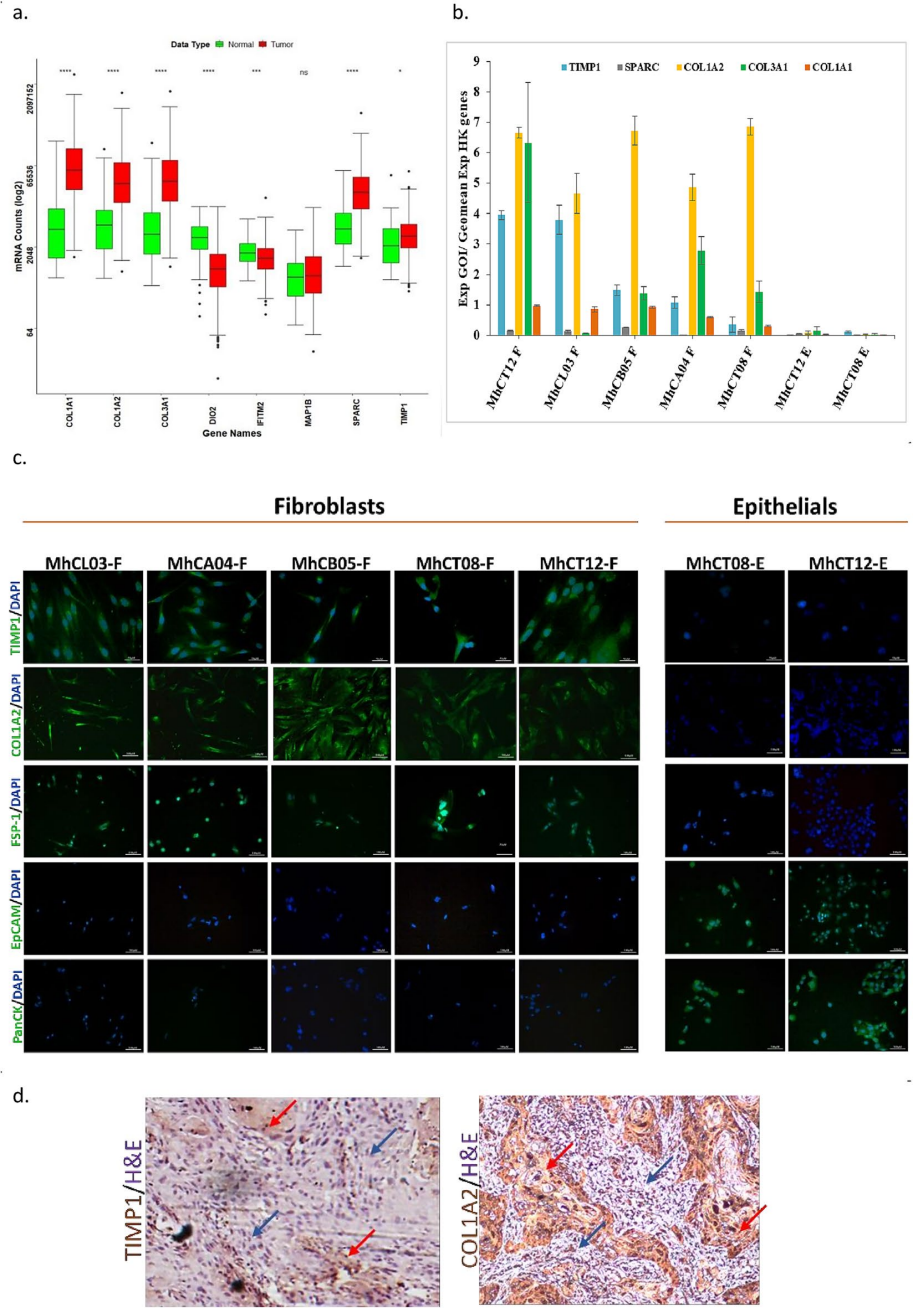
Validation of expression of the candidate CAF markers. (**a**) in TCGA HNSC dataset (*p-value < 0.05; ns—non significant), (**b**) on CAF cells by qPCR; (**c**) by immunofluorescence (40X images) showing staining of TIMP1, COL1A2, FSP-1, EpCAM and PanCK on the indicated cell types; (**d**) Immuno-histochemical validation of TIMP1 and COL1A2 in oral cancer FFPE blocks. Red arrows in the representative image indicate the stromal compartment, while the blue arrows indicate the epithelial compartment of the tissue (images taken at 10X).

Student’s t-test with RNAseq counts from dataset GSE135975 indicated only seven (DIO2, MAP1B, COL1A1, SPARC, COL1A2, COL3A1 and TIMP-1) out of the 22 concordant genes to be upregulated in CAFs over normal fibroblasts. One gene IFITM2 was found to be downregulated (Data not shown). Further using the criterion that a CAF-marker should be over-expressed in tumor samples, by analysis of TCGA HNSC dataset, we selected five genes viz. COL1A1, SPARC, COL1A2, COL3A1 and TIMP-1, where DIO2 and IFITM2 were downregulated and MAP1B did not show much difference (Fig. 2a).

### Validation of putative CAF-marker transcripts

In qPCR analysis of the potential markers, TIMP-1 and COL1A2 were significantly overexpressed (*p* < 0.05) in 5 fibroblasts as compared to epithelial cultures, (Fig. 2b). SPARC, followed by COL1A1, had the lowest mRNA expression among the 5 CAF-markers shortlisted. COL3A1 was found to be significantly overexpressed in four out of five fibroblasts; however in MhCL03-F it was expressed meekly. TIMP-1 and COL1A2 were therefore chosen for further analysis and validation.

### Protein Expression of CAF-markers in cell lines

Protein expression of TIMP-1 and COL1A2 was detected in the fibroblasts but absent in the corresponding epithelial cultures (Fig. 2c). The cells were also qualified by staining with FSP-1, an established fibroblast marker as positive control along with two epithelial specific markers—PanCK and EpCAM as negative control for the CAFs. The fibroblast cells stained only with FSP-1, TIMP-1 and COL1A2 while the epithelial cells stained only with the epithelial specific markers—PanCK and EpCAM (Fig. 2c). Compared to FSP-1, TIMP-1 and COL1A2 displayed uniform staining across the 5 CAFs.

### COL1A2 is the best CAF-marker

Analysis of slides from paraffin embedded oral squamous cell carcinoma blocks using COL1A2 and TIMP-1 staining revealed that TIMP-1, stained meekly (Fig. 2d) as opposed to COL1A2 (Fig. 2d), which showed a strong and significant differential staining in the stromal compartment of oral cancer patient sample, thus winning the race over TIMP-1.

## Discussion

In this study initiating from a data science driven unbiased genome-wide approach, from ~ 400 genes, COL1A2 and TIMP-1 were experimentally validated at the protein level to be the general CAF-markers in HNSC.

Earlier used methods for identification of CAF-markers majorly constituted of immunofluorescence, immunohistochemistry or flow cytometry based individual pre-selected-genecentric techniques. More recently, single cell sequencing based analyses were performed to identify potential CAF signature as a biomarker for the prognosis of HNSC patients, or for the classification of different CAF subtypes; however in these studies, protein level validation of the identified markers was not performed^36,37^. The novel approach described in this study for the identification of CAF-markers is unbiased towards any particular gene, dealing with a large number of in-house and public domain data obtained from completely independent experiments, finally converging at validation at the protein level. This kind of analysis was possible due to the availability of a set of novel CAF cells^43,44^. To our knowledge, the study is the first report of its kind to utilize an integrative way of identifying CAF-markers at mRNA and protein level. The present study identified three collagen markers along with TIMP-1 and SPARC, compared among themselves and with the existing marker, FSP-1 to finally choose COL1A2 followed by TIMP-1.

Collagens are a protein family that helps to build and support numerous structural tissues, including cartilage, bone, tendon, skin, and the white area of the eye (the sclera)^45^. In a study by Misawa et al.^46^, hypermethylation of the COL1A2 promoter occurred with a high frequency and the expression levels of COL1A2 was downregulated in HNSC cell lines, which are epithelial in nature. Collagen has been reported to induce a more aggressive phenotype in HNSC via DDR1^47^. COL1A2 has also been reported extensively to be downregulated in melanoma^48,49^ and bladder cancer^50^, while being upregulated in colorectal cancer^51^, gastric cancer^52,53^, breast cancer^54^ and medulloblastoma^55^. COL1A2 gene codes for a protein that is part of a larger molecule known as type I collagen. The ECM's primary structural component is type I collagen, whose modification has been linked to stromal invasion and epithelial carcinogenesis in several cancers. Mesenchymal fibroblast cells have been characterized by the presence of COL1A2 promoter gene^56^. COL1A1 has been identified as a potential CAF-marker in murine cells^57^. In humans, COL1A2 along with MMP2 and FAP as CAF identifier has been reported in colorectal and pancreatic cancer^13,58,59^. The present study identified three collagen family markers, viz—COL1A2, COL3A1 and COL1A1 as putative CAF identifiers, however, COL1A1 and COL3A1 displayed irregular expression in the five CAFs under study. COL1A2 on the other hand showed an uniform expression among CAFs originating from diverse sources like larynx (MhCL03-F), buccal mucosa (MhCB05-F), upper alveoulus (MhCA04-F) and tongue (MhCT08-F and MhCT12-F)^44^ and hence was taken forward among others. Its function as a CAF factor is yet to be ascertained.

TIMP-1 has been associated with the tumor aggressive phenotype in various cancers^60–62^. Compared to FSP-1, the popular CAF-identifier, both TIMP-1 and COL1A2 displayed uniform and brighter staining across the 5 CAFs tested. However, TIMP-1 did not stain well in IHC on FFPE block, may be due to the difference in TIMP-1 expression across different stages and location of oral cancers^62^.

Though majority of solid tumors, including HNSC, exhibit SPARC expression^63,64^, is linked to EMT signalling^65^ and unfavourable clinico-pathological characteristics^66^, it did not qualify as a dependable marker in the present study, due to its overall lower expression across varieties of CAFs tested.

The study is limited by the fact that all the molecules shortlisted are secretory in nature, hence cannot be used for sorting CAFs from tumors. However, it can be used for their extensive characterization, even by IHC. In the present study we have used golgi-plug to arrest the secretion in the established CAF culture to detect the presence of the putative CAF-markers in cells via immunoflourescence. Curiously, the entire list of the markers identified by the data science driven approach presented here are secretory in nature indicating that the over expressing CAF-factors reach out more effectively to the tumor as well as the other cells of the microenvironment by spreading through the matrix. The CAF-markers open the door to future selective and therapeutically viable targeting of the tumor cells, enabling the establishment of a successful oral cancer therapy regimen.

## Conclusions

The current study shows that COL1A2, followed by TIMP-1, are the most potent CAF-markers found and verified at the transcriptome and protein level across multiple CAF types, differentiating CAF from tumour epithelia and normal fibroblast in HNSC patient samples.

## Methods

**Figure 3:**
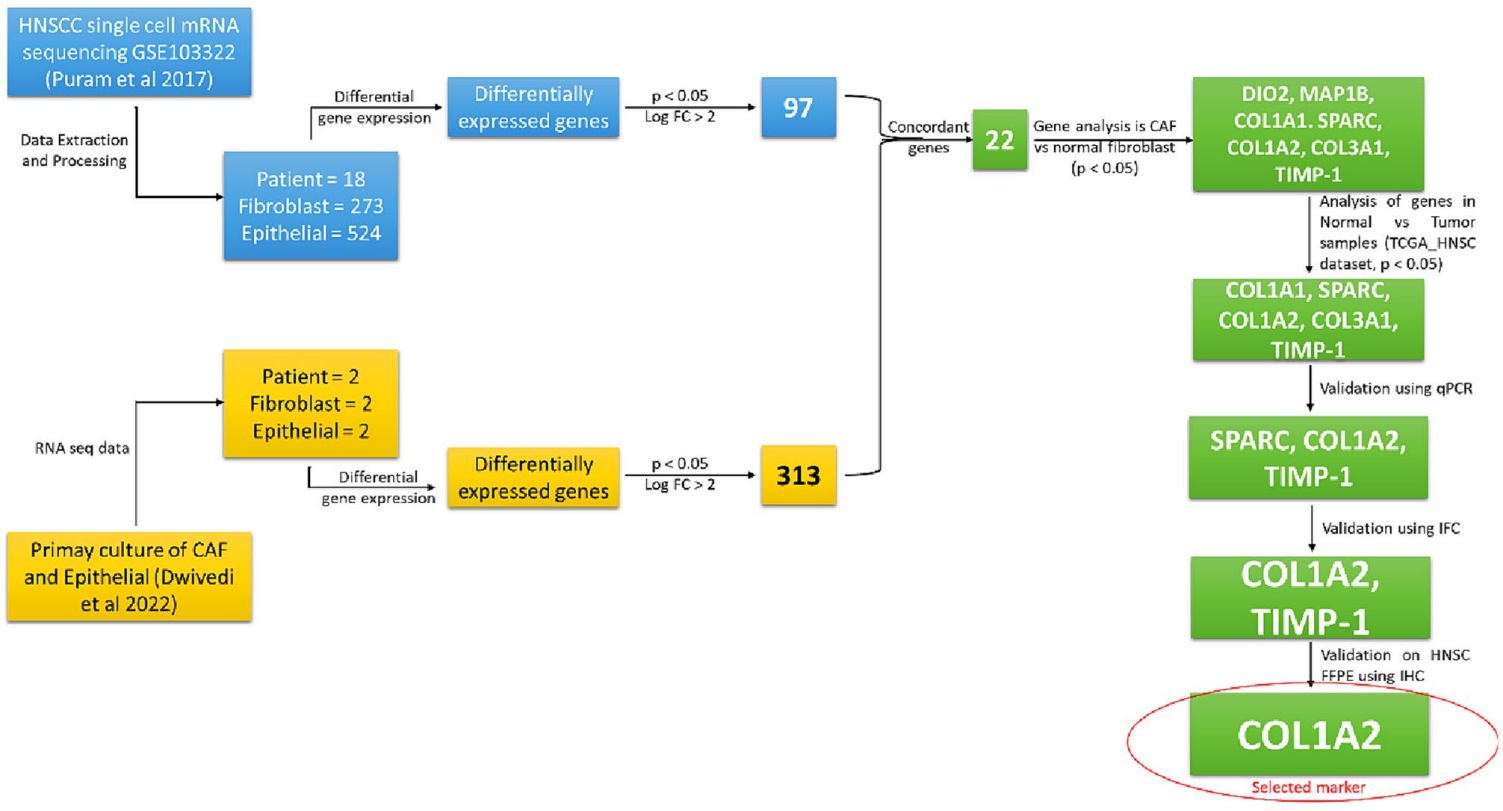
Workflow for identification of putative CAF marker candidates. Data analysis was performed from public databases and in-house RNA-seq data to arrive at a concordant set of differentially expressed genes between epithelial and CAFs which were further selected over normal fibroblasts (Lai et al. 2019), and adjacent normal in TCGA HNSC dataset. The chosen markers were then validated in patient derived CAF cells using qPCR and at protein level by fluorescence microscopy (IFC) and immunohistochemistry (IHC).

The overall study workflow (Fig. 3) begins with patient-derived cell culture maintenance, sequencing, data collection, and ends with final candidate markers using various levels of filters like (i) comparison of CAF and normal fibroblast transcriptome (ii) normal versus tumor transcriptome from a public database, and a final filter of (iii) validation in patient derived samples and FFPE blocks. All methods were performed in accordance with the relevant guidelines and regulations.

### Cell culture

MhCT12-E, MhCT12-F, MhCT08-F, MhCT08-E^43,44^ MhCL03-F, MhCB05-F and MhCA04-F^44^ were maintained RPMI- 1640 media (#AT222A; HiMedia) with 20% Fetal Bovine Serum (FBS; 10,270–106; Gibco), 1X penicillin- streptomycin (#15,140,122; Gibco) and subcultured every 48 h.

### mRNA sequencing of patient derived cell lines

Sequencing of mRNA from the autologous pair—MhCT08-E, MhCT08-F, MhCT12-E and MhCT12-F was performed using Illumina HiSeq series X sequencer using 150 bp paired-end chemistry. Reads obtained from the sequencer were quality-checked and filtered using fastp^67^ and subsequently aligned using STAR^68^ pipeline.

### Acquisition of single cell sequencing data

Search with the terms (((Head and Neck Squamous Cell Carcinoma OR "ORAL CANCER" OR HNSC) AND (single-cell OR "single cell")) with organism filter for homo sapiens in Gene Expression Omnibus (GEO)^69^ yielded three series with accession codes GSE103322, GSE135975 and GSE163872 respectively. GSE103322^36^, single-cell gene expression processed data and GSE135975^47^ RNAseq FPKM (fragments per kilobase of transcript per million mapped reads) values were retrieved from GEO portal. Data from GSE163872 was not considered since the clinical details of the samples were not available.

### Data extraction and processing

For single cell data twenty human gene expression datasets were discovered using primary search terms from the GEO database. GSE103322 was selected appropriately after pruning the data which consisted of sequenced transcriptomes of roughly 6,000 single cells from 18 HNSC patients containing 273 and 524 non-lymphatic fibroblast and epithelial cells. In parallel, RNAseq data from two novel autologous pairs of CAFs and epithelial cells were also analysed.

Differential gene expression (DGE) analysis of these two datasets yielded a set of CAF indicators that were over-expressed in CAFs relative to epithelial cells, which were further assessed for expression in normal fibroblasts and CAFs using RNAseq data from GSE135975^47^, containing 8 patient CAFs and 3 normal fibroblast samples.

### Selection of putative CAF-markers

Over-expressed genes in fibroblasts compared to epithelials in dataset GSE103322 were enumerated using the limma R package. Overexpressed genes with Log Fold Change value ≥ 2 and Benjamini–Hochberg adjusted *p*-value ≤ 0.05 in fibroblasts from the in-house cell line sequencing data and from dataset GSE103322 were combined to identify genes concordant in both differential expression sets. The concordant genes were further filtered for differential expression between cancer-associated and normal fibroblasts (in dataset GSE135975) using t-test to shortlist genes with over-expression in cancer-associated fibroblasts. Expression of these shortlisted genes were assessed in the TCGA HNSC dataset using RNAseq expression values acquired from Broad Institute Firehose portal^70^ to obtain set of genes overexpressed in tumor compared to its normal counterpart.

### RNA isolation, cDNA conversion and qPCR

One million cells were re-suspended and incubated for 10 min on ice in 1 mL of TRIzol^71^ (#15,596,018; Invitrogen) followed by addition of 200µL of chloroform (#496,189; Sigma) and centrifugation at 13,000 g for 15 min at 4˚C. The aqueous layer was transferred into a fresh tube containing equal volume of 100% isopropanol (#DB4DF64078; Merck) without disturbing either the middle interface or the lower organic phase. The tube was gently invert-mixed and incubated at room temperature for 10 min followed by centrifugation at 13,000 *g* for 10 min at 4 °C. The supernatant was discarded and the pellet was washed with 75% ice cold ethanol (#MB228; HiMedia) followed by centrifugation at 7500 g for 5 min at 4 °C. Air-dried pellet was then dissolved in nuclease free water for further processing. For cDNA conversion, 1 μg of total RNA was used in a 20 μL reaction volume with AMV Reverse Transcriptase enzyme (#M0277S; NEB). With a set of three reference genes — TYW5, RIC8B and PLEKHA3^72^, qPCR was performed in triplicates on a Roche LightCycler 480 II instrument using KAPA SyBr green Universal kit (#KK4601; Kapa) in a total of 5 µL reaction volume with 1:5 diluted cDNA and primers (100 nM each). The reaction mix was pre-incubated at 95 °C for 10 s followed by 45 cycles of amplification (95 °C — 1 s; 95 °C — 10 s; 60 °C — 15 s; and 72 °C — 15 s).

RNA was quantified using Qubit RNA Assay BR (#Q10210; Invitrogen) for sequencing on Illumina HiSeq X instrument to generate 30 M, 150 bp paired end reads.

### Design of primers

Primers, with amplicon sizes of 100–150 base pairs and melting temperatures of 60–65 °C were designed using Primer Bank Harvard and synthesized by Eurofins, Bangalore, India (primers used in the study are listed below).

### Primers used for qPCR

**Table:** 

S. No	Gene name	Forward primer (5′-3′)	Reverse primer (5′-3′)
1	TYW5	CAGCATCAAGAGCTGCACAAA	TGTGTAGGACCATTCGTCGTG
2	PLEKHA3	ACTGTGACCTCTTAATGCAGC	CTCAAGCGTTGTGATGAATGTG
3	RIC8B	ATAGTGTTCAACAGTCAGATGGC	GCAAGCGCAAGTCAAAGCA
4	TIMP1	AGAGTGTCTGCGGATACTTCC	CCAACAGTGTAGGTCTTGGTG
5	SPARC	CCCATTGGCGAGTTTGAGAAG	CAAGGCCCGATGTAGTCCA
6	COL1A2	GGCCCTCAAGGTTTCCAAGG	CACCCTGTGGTCCAACAACTC
7	COL3A1	GCCAAATATGTGTCTGTGACTCA	GGGCGAGTAGGAGCAGTTG
8	COL1A1	GTGCGATGACGTGATCTGTGA	CGGTGGTTTCTTGGTCGGT

### qPCR data analysis

For efficiency check a two-fold five-point dilution of Cal27-Parental cDNA was used as template. Thermo primer efficiency calculator^73^ was used to calculate the efficiency of primers as E = 10^-1/slope^. For data analysis, Quantification cycle (Cq) values thus obtained were subtracted by geometric mean of house-keeping genes (HK genes) to obtain ΔCq = Cq_sample_ − Geom mean Cq_HK genes_ from which the relative expression was calculated as E^-ΔCq^ for each replicate.

### Immunofluorescence

Immunofluorescence was performed as detailed previously^43,44^ and in accordance to the protocol by EuroMabNet^74^. Briefly, for analysis by fluorescence, 20,000 cells in 100µL of medium were grown on coverslips and allowed to attach overnight followed by fixing with 100% ice cold methanol (#AS059; HiMedia) for 15 min at -20˚C and blocking in 1% Bovine Serum Albumin (BSA; # TC194; HiMedia) and 0.3% triton-X 100 (#10,655; Fischer Scientific) in Phosphate Buffer Saline (PBS; #10,010–023; Gibco) for 1 h at room temperature. Cells were then incubated with anti-TIMP-1 (MAB970-SP; R&D sysemts, used at 0.5 mg/mL) and anti-COL1A2 (#GTX102996; GeneTex; used at 0.5 mg/mL) along with FSP-1 (#F4771; Sigma; used at 2 mg/mL) and epithelial specific markers PanCK (#4545; CST; used at 1:200 dilution) and EpCAM (#AN820; BioGeneX; working solution) as primary antibodies for 3 h at room temperature, followed by treatment with anti-mouse Alexa488-conjugated secondary antibody (#A11029; Invitrogen; 1:200) for 1 h at room temperature in dark. The processed coverslips were mounted on slides with 4′,6-diamidino-2-phenylindole (DAPI) (#F6057; HiMedia) histology mount and visualized under Zeiss Scope A1 fluorescent microscope. All the intermittent washes were given thrice with 1X PBS.

### Immunohistochemistry

FFPE tumor blocks (n = 10) containing both the tumor stroma and tumor epithelia from oral cancer patients with lesion of buccal mucosa were used for immunohistochemical staining of COL1A2 and TIMP-1. Briefly, slides were deparaffinized for 30 min at 70 °C and passedthrough xylene, graded alcohol, and deionized water. Slides were then rinsed in low pH antigen retreival buffer, and microwaved at 60% power. After cooling, the slides were incubated in 3% hydrogen peroxide (#1,072,090,500; Supelco) for 10 min followed by rinsing with TBST and blocking for 30 min using 3% BSA and incubated with respective antibodies at room temperature for 60 min followed by rinsing with TBST and incubation with DAKO kit (#K5007; Dako; Agilent Technologies) for 30 min. Signal was detected using DAB followed by counterstain with hematoxylin solution. Slides were washed with xylene before being mounted using DPX mountant (#DAL1025; Qualigen Fine Chemicals TM; ThermoFisher Scientific) and observed under Nikon bright field microscope.

### Ethical approval

The present study was approved [NHH/MEC-CL-EA-2015-405(A)] by the ethics committee of Narayana Health (Bangalore, India). Informed consent for the study was obtained from all patients.

### Supplementary Information

Supplementary Information.

## Data Availability

The datasets generated and/or analysed during the current study are available in GEO repository with the accession number—GSE233043. All the materials used have been enlisted in supplementary table IV.

## Abbreviations

ACTA2: Actin Alpha 2
AKT: Alpha serine/threonine-protein kinase
BSA: Bovine serum albumin
CAF: Cancer associated fibroblast
CAV1: Caveolin 1
cDNA: Complementary deoxyribonucleic acid
COL11A: Collagen type XI Alpha 1 chain
COL1A1: Collagen type I Alpha 1 chain
COL1A2: Collagen type I Alpha 2 chain
COL3A1: Collagen type III Alpha 1 chain
CRC: Colorectal cancer
CXCR4: C-X-C Motif chemokine receptor 4
DAPI: 4′,6-Diamidino-2-phenylindole
ddCT: Delta-delta Ct
DGE: Differential gene expression
DIO2: Iodothyronine Deiodinase 2
ECM: Extracellular matrix
EMT: Epithelial to mesenchymal transition
EpCAM: Epithelial cellular adhesion molecule
EREG: Epiregulin
FACS: Flourescence associated cell sorting
FAP: Fibroblast-activation protein
FBS: Fetal Bovine serum
FGF7: Fibroblast growth factor 7
FSP1: Fibroblast-specific protein-1
HGF: Hepatocyte growth factor
HNSC: Head and neck squamous cell carcinoma
iCAFs: Inflammatory CAFs
IL-1β: Interleukin 1 Beta
ITGA11: Integrin subunit Alpha 11
MAP1B: Microtubule associated protein 1B
MFAP5: Microfibril associated protein 5
MMP11: Matrix Metallopeptidase 11
myCAFs: Myofibroblastic CAFs
NCBI: National Center for Biotechnology Information.
NOX4: NADPH oxidase 4
OSCC: Oral squamous cell carcinoma
PBS: Phosphate buffer saline
PDAC: Pancreatic ductal adenocarcinoma
PDGFRα: Platelet derived growth factor receptor alpha
PDGFRβ: Platelet Derived growth factor receptor beta
PDPN: Podoplanin
PI3K: Phosphatidylinositol-4,5-bisphosphate 3-kinase
PLEKHA3: Pleckstrin homology domain containing A3
POSTN: Periostin
qPCR: Quantitative polymerase chain reaction
RIC8B: Resistance to inhibitors of cholinesterase 8 Homolog B
RNA: Ribonucleic acid
ROS: Reactive oxygen species
SDF-1: Stromal cell-derived factor-1
SLCC: Stem like cancer cells
SMA: Smooth muscle actin
SMTN: Smoothelin
SPARC: Secreted protein acidic and cysteine rich
STRING: Search tool for the retrieval of interacting genes/proteins
TAGLN: Transgelin
TCGA: The cancer genome atlas
TGF: Transforming growth factor
TIMP-1: Tissue inhibitor of metalloproteinases 1
TME: Tumor Microenvironment
TYW5: TRNA-YW synthesizing protein 5
VIM: Vimentin
