# Supplementary material for: Novel CAF-identifiers via transcriptomic and protein level analysis in HNSC patients
Source: Sci Rep. 2023 Aug 25;13:13899. doi: 10.1038/s41598-023-40908-w (PMC10457345; doi:10.1038/s41598-023-40908-w)
Supplement: Supplementary file 1 — Supplementary Information. [file 41598_2023_40908_MOESM1_ESM.docx]

Supplementary Table I: CAF markers in head and neck cancer

| S.No | Marker | Techniques used | Drawbacks of the marker |
| --- | --- | --- | --- |
| 1. | Vimentin | IHC (1–7), Microscopy (3,8–10), Western blotting (9–15), Immunofluorescence (10–12,14,16–18), qRT-PCR (5,16,18),  Immunocytochemistry (15), Single Cell Sequencing (19) | Vimentin has ubiquitous expression in the whole fibroblast population as well as multiple other cell types, including macrophages, adipocytes, and the cells undergoing EMT severely limits its utility as a CAF-specific marker (20–22). |
| 2. | ACTA2 | IHC (3–7,23–29), Microscopy (3,9), Western blotting (9,11,12,14,15,25,30–32), Immunofluorescence (11,14,16–18,23,24,31,33–35), qRT-PCR (5,16,18,24,28,30,31), Immunocytochemistry (15,36), Single Cell Sequencing (19), Flow Cytometry (25) | ACTA2 displays fluctuating expression amongst various CAF subtypes (37,38). Additionally, the intracellular location of ACTA2 makes it unsuitable for flow-sorting CAF populations. |
| 3. | FAP | Western blotting (12,30), Immunofluorescence (12,33), qRT-PCR (30), Single Cell Sequencing (19) | Epithelial cells undergoing epithelial-mesenchymal transition (EMT) also exhibit higher levels of FAP (39) |
| 4. | FN1 | IHC (4), Western blotting(12), Immunofluorescence (12), Single Cell Sequencing (19) | Recognizes only myofibroblasts (40) |
| 5. | S100A4/FSP1 | IHC (4,23), Immunofluorescence (23), qRT-PCR (41), Flow Cytometry (41) | Varies among subtypes as reported in colorectal cancer (38). |
| 6. | THY1 | Immunofluorescence (16), qRT-PCR (16), Single Cell Sequencing (19) | THY1+ fibroblasts are mainly of the reticular lineage (40) |
| 7. | SHH Ligand and GLI1 | IHC, Immunofluorescence (23) | No reports suggesting its absence in epithelial cells, whereas it is present in the epithelial cell development of epidermis, touch dome, hair, sebaceous gland, mammary gland, tooth, nail, gastric epithelium, and intestinal epithelium (42) |
| 8. | COL8A1 and COL11A1 | RNA seq (43) | Not enough experimental validation reports |
| 9. | PDPN, CTGF, JUN, FOS, FGF7, FGF7, TGFBR2/3, MMP11, CAV1, FN1, MMP2, JUNB, IER2, IL8, FOSL1, STAT1, IDO1, ALDH1A1, ALDH3A1, GSTM1, GSTA1, CYP4F11, CYP4F3, ABCC1, GPX2, GSTM2, GSTM3, GSTM4, MYC, TNF, S100A9, PDGFRA, PDGFRL, FTH1, TM4SF1, SLC16A3, IER3 | Single Cell Sequencing (19,44) | Transgelin (TAGLN) and Periostin (POSTN) (45) in CAFs vary among subtypes, Podoplanin (PDPN), a membrane-bound marker that has been utilised to identify pro-tumorigenic fibroblast subpopulations lacks specificity since it is also expressed in epithelial tumor cells and inflammatory macrophages (46,47). Neither PDGFRα nor PDGFRβ are significantly upregulate in CAF populations, due to a more stable expression which is not sensitive to environmental variables like hypoxia, both have been considered as CAF markers in breast cancer (48). For all the markers described, there is not enough experimental validation reports. |

Supplementary table II: Techniques for identification of CAF markers: pan-cancer

| S.No | Techniques used | Markers | Cancer type |
| --- | --- | --- | --- |
| 1 | Immunohistochemistry | FAP (49), MFAP5 (50), COL11A1 (51), TN-C (52), PDPN (53), ITGA11 (28), POSTN (54) | Melanocytic skin tumors, Ovarian Cancer, Pancreatic cancer, Prostate cancer, Melanoma, Head and Neck Squamous Cell Carcinoma, Bladder cancer |
| 2 | Immunofluorescence | ACTA2 (55), Vimentin (56), S100A4 (57) | Breast cancer, Human colon tumors, Melanoma |
| 3 | Western blotting | MFAP5 (50) | Ovarian cancer |
| 4 | qRT-PCR | ITGA11 (28) | Head and Neck Squamous Cell Carcinoma |
| 5 | Flow cytometry | PDGFR alpha and Beta (58), S100A4 (57) | Breast cancer |
| 6 | Immunocytochemistry | Vimentin (56) | Human colon tumors |
| 7 | single cell sequencing | ACTA2, FAP, PDPN, CTGF, JUN, FOS, FGF7, VIM, THY1, FGF7, TGFBR2/3, MMP11, CAV1, FN1, MMP2, JUNB, IER2, IL8, FOSL1, STAT1, IDO1, ALDH1A1, ALDH3A1, GSTM1, GSTA1, CYP4F11, CYP4F3, ABCC1, GPX2, GSTM2, GSTM3, GSTM4, MYC, TNF, S100A9, PDGFRA, PDGFRL (19)  BGN, LUM, CCL19, CEBPD, and ID3 (59)  ADAMTSL2, SLCO2A1, CD4, HMGXB3, GCN1, and LUC7L3 (60) | Head and Neck Squamous Cell Carcinoma, Breast Cancer |

Supplementary Table III. P-Value for the selected marker genes in CAF Versus Fibroblast data using t.test.

| **HGNC** | **Difference between the counts of CAF and Fibroblast** | t-test p-value |
| --- | --- | --- |
| DIO2 | 4270.458 | 0.000443 |
| MAP1B | 42710.75 | 0.001093 |
| IFITM2 | -495.833 | 0.001899 |
| COL1A1 | 127671.2 | 0.008181 |
| SPARC | 60279.13 | 0.015402 |
| COL1A2 | 192046.8 | 0.019645 |
| COL3A1 | 119438.3 | 0.022073 |
| TIMP1 | 10349.42 | 0.023561 |

Supplementary Table IV Clinical and pathological details of the FFPE blocks.

| **Patient No.** | **Patient Age (years)/ sex** | **Primary Site** | **Pathological Staging** | **Clinical staging** |
| --- | --- | --- | --- | --- |
| 1 | 75/F | Buccal Mucosa | pT3N2Mx | cT3aN2b |
| 2 | 64/F | Tongue | pT3N3bM1 | cT4cN3 |
| 3 | 33/F | Tongue | pT4N1aM0 | cT3aN2c |
| 4 | 59/F | Larynx | pT4N3cMx | cT4aN3a |
| 5 | 76/M | Tongue | pT2N2bM2 | cT2aN2c |
| 6 | 69/M | Tongue | pT4N3aM2 | cT4aN2c |
| 7 | 72/M | Larynx | pT4N1bMx | cT4aN2a |
| 8 | 58/M | Larynx | pT3N1cM0 | cT4aN1c |
| 9 | 38/M | Buccal Mucosa | pT3N2aM2 | cT4aN2a |
| 10 | 29/M | Buccal Mucosa | pT2N0M0 | cT2aN0 |

**References**

1. Rosenthal E, McCrory A, Talbert M, Young G, Murphy-Ullrich J, Gladson C. Elevated expression of TGF-beta1 in head and neck cancer-associated fibroblasts. Mol Carcinog. 2004 Jun;40(2):116–21.

2. Rosenthal EL, McCrory A, Talbert M, Carroll W, Magnuson JS, Peters GE. Expression of proteolytic enzymes in head and neck cancer-associated fibroblasts. Arch Otolaryngol Head Neck Surg. 2004 Aug;130(8):943–7.

3. Liu Y, Hu T, Shen J, Li SF, Lin JW, Zheng XH, et al. Separation, cultivation and biological characteristics of oral carcinoma-associated fibroblasts. Oral Dis. 2006 Jul;12(4):375–80.

4. Dourado RC, Porto LPA, Leitão ÁCGH, Cerqueira PSG, Dos Santos JN, Ramalho LMP, et al. Immunohistochemical Characterization of Cancer-associated Fibroblasts in Oral Squamous Cell Carcinoma. Appl Immunohistochem Mol Morphol AIMM [Internet]. 2018 Oct 1 [cited 2022 Aug 17];26(9):640–7. Available from: https://pubmed.ncbi.nlm.nih.gov/28968269/

5. Kartha VK, Stawski L, Han R, Haines P, Gallagher G, Noonan V, et al. PDGFRβ is a novel marker of stromal activation in oral squamous cell carcinomas. PLoS One. 2016 Apr;11(4).

6. Bian L, Sun X, Jin K, He Y. Oral cancer-associated fibroblasts inhibit heat-induced apoptosis in Tca8113 cells through upregulated expression of Bcl-2 through the Mig/CXCR3 axis. Oncol Rep [Internet]. 2012 Dec [cited 2022 Aug 17];28(6):2063–8. Available from: https://pubmed.ncbi.nlm.nih.gov/22961626/

7. Jung DW, Che ZM, Kim J, Kim K, Kim KY, Williams D, et al. Tumor-stromal crosstalk in invasion of oral squamous cell carcinoma: a pivotal role of CCL7. Int J cancer [Internet]. 2010 Jul 15 [cited 2022 Aug 17];127(2):332–44. Available from: https://pubmed.ncbi.nlm.nih.gov/19937793/

8. New J, Arnold L, Ananth M, Alvi S, Thornton M, Werner L, et al. Secretory Autophagy in Cancer-Associated Fibroblasts Promotes Head and Neck Cancer Progression and Offers a Novel Therapeutic Target. Cancer Res. 2017 Dec;77(23):6679–91.

9. T J, T Y, J L, Y M, L F, A E-N, et al. Brain derived neutrophic factor (BDNF) coordinates lympho-vascular metastasis through a fibroblast-governed paracrine axis in the tumor microenvironment. Cancer cell Microenviron [Internet]. 2017 Jul 10 [cited 2022 Aug 17];4(2). Available from: https://pubmed.ncbi.nlm.nih.gov/28966935/

10. Zhang Z, Tao D, Zhang P, Liu X, Zhang Y, Cheng J, et al. Hyaluronan synthase 2 expressed by cancer-associated fibroblasts promotes oral cancer invasion. J Exp Clin Cancer Res [Internet]. 2016 Nov 25 [cited 2022 Aug 17];35(1). Available from: https://pubmed.ncbi.nlm.nih.gov/27884164/

11. Wang Y, Jing Y, Ding L, Zhang X, Song Y, Chen S, et al. Epiregulin reprograms cancer-associated fibroblasts and facilitates oral squamous cell carcinoma invasion via JAK2-STAT3 pathway. J Exp Clin Cancer Res [Internet]. 2019 Jun 24 [cited 2022 Aug 17];38(1). Available from: https://pubmed.ncbi.nlm.nih.gov/31234944/

12. Wang Q, Zhang YC, Zhu LF, Pan L, Yu M, Shen WL, et al. Heat shock factor 1 in cancer-associated fibroblasts is a potential prognostic factor and drives progression of oral squamous cell carcinoma. Cancer Sci [Internet]. 2019 May 1 [cited 2022 Aug 17];110(5):1790–803. Available from: https://pubmed.ncbi.nlm.nih.gov/30843645/

13. Yu B, Wu K, Wang X, Zhang J, Wang L, Jiang Y, et al. Periostin secreted by cancer-associated fibroblasts promotes cancer stemness in head and neck cancer by activating protein tyrosine kinase 7. Cell Death Dis [Internet]. 2018 Nov 1 [cited 2022 Aug 17];9(11). Available from: https://pubmed.ncbi.nlm.nih.gov/30348980/

14. Qin X, Yan M, Wang X, Xu Q, Wang X, Zhu X, et al. Cancer-associated Fibroblast-derived IL-6 Promotes Head and Neck Cancer Progression via the Osteopontin-NF-kappa B Signaling Pathway. Theranostics. 2018;8(4):921–40.

15. Chen SF, Nieh S, Jao SW, Wu MZ, Liu CL, Chang YC, et al. The paracrine effect of cancer-associated fibroblast-induced interleukin-33 regulates the invasiveness of head and neck squamous cell carcinoma. J Pathol [Internet]. 2013 Oct [cited 2022 Aug 17];231(2):180–9. Available from: https://pubmed.ncbi.nlm.nih.gov/23775566/

16. Oppel F, Shao S, Schürmann M, Goon P, Albers AE, Sudhoff H. An Effective Primary Head and Neck Squamous Cell Carcinoma In Vitro Model. Cells [Internet]. 2019 Jun 7 [cited 2021 Oct 5];8(6):555. Available from: /pmc/articles/PMC6628367/

17. Wei LY, Lee JJ, Yeh CY, Yang CJ, Kok SH, Ko JY, et al. Reciprocal activation of cancer-associated fibroblasts and oral squamous carcinoma cells through CXCL1. Oral Oncol [Internet]. 2019 Jan 1 [cited 2022 Aug 17];88:115–23. Available from: https://pubmed.ncbi.nlm.nih.gov/30616781/

18. Li Y-Y, Zhou C-X, Gao Y. Interaction between oral squamous cell carcinoma cells and fibroblasts through TGF-β1 mediated by podoplanin. Exp Cell Res. 2018 Aug;369(1):43–53.

19. Puram S V., Tirosh I, Parikh AS, Patel AP, Yizhak K, Gillespie S, et al. Single-Cell Transcriptomic Analysis of Primary and Metastatic Tumor Ecosystems in Head and Neck Cancer. Cell [Internet]. 2017 Dec 14 [cited 2022 Feb 21];171(7):1611-1624.e24. Available from: https://pubmed.ncbi.nlm.nih.gov/29198524/

20. Gascard P, Tlsty TD. Carcinoma-associated fibroblasts: orchestrating the composition of malignancy. Genes Dev [Internet]. 2016 May 1 [cited 2022 Jul 7];30(9):1002–19. Available from: https://pubmed.ncbi.nlm.nih.gov/27151975/

21. Hsia LT, Ashley N, Ouaret D, Wang LM, Wilding J, Bodmer WF. Myofibroblasts are distinguished from activated skin fibroblasts by the expression of AOC3 and other associated markers. Proc Natl Acad Sci U S A [Internet]. 2016 Apr 12 [cited 2022 Jul 7];113(15):E2162–71. Available from: https://pubmed.ncbi.nlm.nih.gov/27036009/

22. Kalluri R, Weinberg RA. The basics of epithelial-mesenchymal transition. J Clin Invest [Internet]. 2009 Jun 6 [cited 2022 Jul 7];119(6):1420. Available from: /pmc/articles/PMC2689101/

23. Guimaraes VSN, Vidal MTA, de Faro Valverde L, de Oliveira MG, de Oliveira Siquara da Rocha L, Coelho PLC, et al. Hedgehog pathway activation in oral squamous cell carcinoma: cancer-associated fibroblasts exhibit nuclear GLI-1 localization. J Mol Histol. 2020 Dec;51(6):675–84.

24. Al-Magsoosi MJN, Lambert DW, Ali Khurram S, Whawell SA. Oral cancer stem cells drive tumourigenesis through activation of stromal fibroblasts. Oral Dis. 2021 Sep;27(6):1383–93.

25. Sun LP, Xu K, Cui J, Yuan DY, Zou B, Li J, et al. Cancer‑associated fibroblast‑derived exosomal miR‑382‑5p promotes the migration and invasion of oral squamous cell carcinoma. Oncol Rep [Internet]. 2019 [cited 2022 Aug 17];42(4):1319–28. Available from: https://pubmed.ncbi.nlm.nih.gov/31364748/

26. Dourado MR, de Oliveira CE, Sawazaki-Calone I, Sundquist E, Coletta RD, Salo T. Clinicopathologic significance of ROCK2 expression in oral squamous cell carcinomas. J Oral Pathol Med [Internet]. 2018 Feb 1 [cited 2022 Aug 17];47(2):121–7. Available from: https://pubmed.ncbi.nlm.nih.gov/29052912/

27. Elmusrati AA, Pilborough AE, Khurram SA, Lambert DW. Cancer-associated fibroblasts promote bone invasion in oral squamous cell carcinoma. Br J Cancer [Internet]. 2017 Sep 5 [cited 2022 Aug 17];117(6):867–75. Available from: https://pubmed.ncbi.nlm.nih.gov/28742795/

28. Parajuli H, Teh MT, Abrahamsen S, Christoffersen I, Neppelberg E, Lybak S, et al. Integrin α11 is overexpressed by tumour stroma of head and neck squamous cell carcinoma and correlates positively with alpha smooth muscle actin expression. J Oral Pathol Med [Internet]. 2017 Apr 1 [cited 2022 Aug 17];46(4):267–75. Available from: https://pubmed.ncbi.nlm.nih.gov/27699902/

29. Fujii N, Shomori K, Shiomi T, Nakabayashi M, Takeda C, Ryoke K, et al. Cancer-associated fibroblasts and CD163-positive macrophages in oral squamous cell carcinoma: their clinicopathological and prognostic significance. J Oral Pathol Med [Internet]. 2012 Jul [cited 2022 Aug 17];41(6):444–51. Available from: https://pubmed.ncbi.nlm.nih.gov/22296275/

30. Choi S-Y, Oh SY, Kang SH, Kang S-M, Kim J, Lee H-J, et al. NAB 2-Expressing Cancer-Associated Fibroblast Promotes HNSCC Progression. Cancers (Basel). 2019 Mar;11(3).

31. Dourado MR, Korvala J, Åström P, De Oliveira CE, Cervigne NK, Mofatto LS, et al. Extracellular vesicles derived from cancer-associated fibroblasts induce the migration and invasion of oral squamous cell carcinoma. J Extracell vesicles [Internet]. 2019 Jan 1 [cited 2022 Aug 17];8(1). Available from: https://pubmed.ncbi.nlm.nih.gov/30788085/

32. Wu MH, Hong HC, Hong TM, Chiang WF, Jin YT, Chen YL. Targeting galectin-1 in carcinoma-associated fibroblasts inhibits oral squamous cell carcinoma metastasis by downregulating MCP-1/CCL2 expression. Clin Cancer Res [Internet]. 2011 Mar 15 [cited 2022 Aug 17];17(6):1306–16. Available from: https://pubmed.ncbi.nlm.nih.gov/21385934/

33. Kang SH, Oh SY, Lee H-J, Kwon T-G, Kim J-W, Lee S-T, et al. Cancer-Associated Fibroblast Subgroups Showing Differential Promoting Effect on HNSCC Progression. Cancers (Basel). 2021 Feb;13(4).

34. Kwa MQ, Herum KM, Brakebusch C. Cancer-associated fibroblasts: how do they contribute to metastasis? Clin Exp Metastasis. 2019 Apr;36(2):71–86.

35. Wheeler SE, Shi H, Lin F, Dasari S, Bednash J, Thorne S, et al. Enhancement of head and neck squamous cell carcinoma proliferation, invasion, and metastasis by tumor-associated fibroblasts in preclinical models. Head Neck [Internet]. 2014 Mar [cited 2022 Aug 17];36(3):385–92. Available from: https://pubmed.ncbi.nlm.nih.gov/23728942/

36. Fullár A, Kovalszky I, Bitsche M, Romani A, Schartinger VH, Sprinzl GM, et al. Tumor cell and carcinoma-associated fibroblast interaction regulates matrix metalloproteinases and their inhibitors in oral squamous cell carcinoma. Exp Cell Res [Internet]. 2012 Aug 1 [cited 2022 Aug 17];318(13):1517–27. Available from: https://pubmed.ncbi.nlm.nih.gov/22516051/

37. Latif N, Sarathchandra P, Chester AH, Yacoub MH. Expression of smooth muscle cell markers and co-activators in calcified aortic valves. Eur Heart J [Internet]. 2015 Jun 1 [cited 2022 Jul 6];36(21):1335–45. Available from: https://pubmed.ncbi.nlm.nih.gov/24419809/

38. Li H, Courtois ET, Sengupta D, Tan Y, Chen KH, Goh JJL, et al. Reference component analysis of single-cell transcriptomes elucidates cellular heterogeneity in human colorectal tumors. Nat Genet [Internet]. 2017 May 1 [cited 2022 Jul 6];49(5):708–18. Available from: https://pubmed.ncbi.nlm.nih.gov/28319088/

39. Kahounová Z, Kurfürstová D, Bouchal J, Kharaishvili G, Navrátil J, Remšík J, et al. The fibroblast surface markers FAP, anti-fibroblast, and FSP are expressed by cells of epithelial origin and may be altered during epithelial-to-mesenchymal transition. Cytometry A [Internet]. 2018 Sep 1 [cited 2022 Jul 6];93(9):941–51. Available from: https://pubmed.ncbi.nlm.nih.gov/28383825/

40. Liu T, Zhou L, Li D, Andl T, Zhang Y. Cancer-associated fibroblasts build and secure the tumor microenvironment. Front Cell Dev Biol. 2019;7(APR):60.

41. N D, G C, V P, MA K, A S, M D. Establishment and characterization of novel autologous pair primary cultures from two Indian non-habitual tongue carcinoma patients. 2022 Jan;

42. Zheng L, Rui C, Zhang H, Chen J, Jia X, Xiao Y. Sonic hedgehog signaling in epithelial tissue development. Regen Med Res [Internet]. 2019 [cited 2022 Aug 17];7:3. Available from: /pmc/articles/PMC6941452/

43. Lai SL, Tan ML, Hollows RJ, Robinson M, Ibrahim M, Margielewska S, et al. Collagen Induces a More Proliferative, Migratory and Chemoresistant Phenotype in Head and Neck Cancer via DDR1. Cancers (Basel). 2019 Nov;11(11).

44. Yang Y, Ma B, Han L, Xu W, Du X, Wei W, et al. Integrated single-cell and bulk RNA sequencing analyses reveal a prognostic signature of cancer-associated fibroblasts in head and neck squamous cell carcinoma. Front Genet [Internet]. 2022 Dec 8 [cited 2023 Apr 21];13. Available from: /pmc/articles/PMC9775281/

45. Planche A, Bacac M, Provero P, Fusco C, Delorenzi M, Stehle JC, et al. Identification of Prognostic Molecular Features in the Reactive Stroma of Human Breast and Prostate Cancer. PLoS One [Internet]. 2011 [cited 2022 Jul 7];6(5):e18640. Available from: https://journals.plos.org/plosone/article?id=10.1371/journal.pone.0018640

46. Kerrigan AM, Navarro-Nuñez L, Pyz E, Finney BA, Willment JA, Watson SP, et al. Podoplanin-expressing inflammatory macrophages activate murine platelets via CLEC-2. J Thromb Haemost [Internet]. 2012 Mar [cited 2022 Jul 7];10(3):484–6. Available from: https://pubmed.ncbi.nlm.nih.gov/22212362/

47. Atsumi N, Ishii G, Kojima M, Sanada M, Fujii S, Ochiai A. Podoplanin, a novel marker of tumor-initiating cells in human squamous cell carcinoma A431. Biochem Biophys Res Commun [Internet]. 2008 Aug 15 [cited 2022 Jul 7];373(1):36–41. Available from: https://pubmed.ncbi.nlm.nih.gov/18539139/

48. Madsen CD, Pedersen JT, Venning FA, Singh LB, Moeendarbary E, Charras G, et al. Hypoxia and loss of PHD2 inactivate stromal fibroblasts to decrease tumour stiffness and metastasis. EMBO Rep [Internet]. 2015 Oct [cited 2022 Jul 6];16(10):1394–408. Available from: https://pubmed.ncbi.nlm.nih.gov/26323721/

49. Park JE, Lenter MC, Zimmermann RN, Garin-Chesa P, Old LJ, Rettig WJ. Fibroblast activation protein, a dual specificity serine protease expressed in reactive human tumor stromal fibroblasts. J Biol Chem [Internet]. 1999 Dec 17 [cited 2022 Aug 17];274(51):36505–12. Available from: https://pubmed.ncbi.nlm.nih.gov/10593948/

50. Leung CS, Yeung TL, Yip KP, Pradeep S, Balasubramanian L, Liu J, et al. Calcium-dependent FAK/CREB/TNNC1 signalling mediates the effect of stromal MFAP5 on ovarian cancer metastatic potential. Nat Commun [Internet]. 2014 [cited 2022 Aug 17];5. Available from: https://pubmed.ncbi.nlm.nih.gov/25277212/

51. García-Pravia C, Galván JA, Gutiérrez-Corral N, Solar-García L, García-Pérez E, García-Ocaña M, et al. Overexpression of COL11A1 by cancer-associated fibroblasts: clinical relevance of a stromal marker in pancreatic cancer. PLoS One [Internet]. 2013 Oct 23 [cited 2022 Aug 17];8(10). Available from: https://pubmed.ncbi.nlm.nih.gov/24194920/

52. Ni WD, Yang ZT, Cui CA, Cui Y, Fang LY, Xuan YH. Tenascin-C is a potential cancer-associated fibroblasts marker and predicts poor prognosis in prostate cancer. Biochem Biophys Res Commun [Internet]. 2017 May 6 [cited 2022 Aug 17];486(3):607–12. Available from: https://pubmed.ncbi.nlm.nih.gov/28341124/

53. Kan S, Konishi E, Arita T, Ikemoto C, Takenaka H, Yanagisawa A, et al. Podoplanin expression in cancer-associated fibroblasts predicts aggressive behavior in melanoma. J Cutan Pathol [Internet]. 2014 [cited 2022 Aug 17];41(7):561–7. Available from: https://pubmed.ncbi.nlm.nih.gov/24588302/

54. Silvers CR, Liu YR, Wu CH, Miyamoto H, Messing EM, Lee YF. Identification of extracellular vesicle-borne periostin as a feature of muscle-invasive bladder cancer. Oncotarget [Internet]. 2016 Apr 26 [cited 2022 Aug 17];7(17):23335–45. Available from: https://pubmed.ncbi.nlm.nih.gov/26981774/

55. Lazard D, Sastre X, Frid MG, Glukhova MA, Thiery JP, Koteliansky VE. Expression of smooth muscle-specific proteins in myoepithelium and stromal myofibroblasts of normal and malignant human breast tissue. Proc Natl Acad Sci U S A [Internet]. 1993 [cited 2022 Aug 17];90(3):999–1003. Available from: https://pubmed.ncbi.nlm.nih.gov/8430113/

56. Herrera M, Islam ABMMK, Herrera A, Martín P, García V, Silva J, et al. Functional heterogeneity of cancer-associated fibroblasts from human colon tumors shows specific prognostic gene expression signature. Clin Cancer Res [Internet]. 2013 Nov 1 [cited 2022 Aug 17];19(21):5914–26. Available from: https://pubmed.ncbi.nlm.nih.gov/24052018/

57. Grum-Schwensen B, Klingelhofer J, Berg CH, El-Naaman C, Grigorian M, Lukanidin E, et al. Suppression of tumor development and metastasis formation in mice lacking the S100A4(mts1) gene. Cancer Res [Internet]. 2005 May 1 [cited 2022 Aug 17];65(9):3772–80. Available from: https://pubmed.ncbi.nlm.nih.gov/15867373/

58. Sharon Y, Alon L, Glanz S, Servais C, Erez N. Isolation of normal and cancer-associated fibroblasts from fresh tissues by Fluorescence Activated Cell Sorting (FACS). J Vis Exp [Internet]. 2013 Jan 14 [cited 2022 Aug 17];(71). Available from: https://pubmed.ncbi.nlm.nih.gov/23354290/

59. Hu J, Jiang Y, Wei Q, Li B, Xu S, Wei G, et al. Development of a Cancer-Associated Fibroblast-Related Prognostic Model in Breast Cancer via Bulk and Single-Cell RNA Sequencing. Biomed Res Int [Internet]. 2022 [cited 2022 Dec 30];2022. Available from: /pmc/articles/PMC9735320/

60. Yu L, Shen N, Shi Y, Shi X, Fu X, Li S, et al. Characterization of cancer-related fibroblasts (CAF) in hepatocellular carcinoma and construction of CAF-based risk signature based on single-cell RNA-seq and bulk RNA-seq data. Front Immunol [Internet]. 2022 Sep 23 [cited 2022 Dec 30];13:1009789. Available from: /pmc/articles/PMC9537943/
